# Dissolution of Pre-Existing Platelet Thrombus by Synergistic Administration of Low Concentrations of Bifunctional Antibodies against β3 Integrin

**DOI:** 10.1371/journal.pone.0027012

**Published:** 2011-10-28

**Authors:** Suying Dang, Tao Hong, Thomas Wisniewski, Wei Zhang

**Affiliations:** 1 Department of Medical Genetics, Shanghai Jiao Tong University School of Medicine, Shanghai, People's Republic of China; 2 Institutes for Advanced Interdisciplinary Research, East China Normal University, Shanghai, People's Republic of China; 3 Departments of Neurology, Pathology and Psychiatry, New York University School of Medicine, New York, New York, United States of America; Heart Center Munich, Germany

## Abstract

Most antithrombotic approaches target prevention rather than the more clinically relevant issue of resolution of an existing thrombus. In this study, we describe a novel and effective therapeutic strategy for ex vivo clearance of pre-existing platelet thrombus by the combination of two bifunctional platelet GPIIIa49-66 ligands that target different parts of the arterial thrombus. We produced an additional GPIIIa49-66 agent (named APAC), which homes to activated platelets. Like our previously described SLK (which targets newly deposited fibrin strands surrounding the platelet thrombus), APAC destroys platelet aggregates ex vivo in an identical fashion with 85% destruction of platelet aggregates at 2 hours. The combined application of APAC and SLK demonstrated a ∼2 fold greater platelet thrombus dissolution than either agent alone at a low concentration (0.025 µM). Platelet-rich clot lysis experiments demonstrated the time required for 50% platelet-rich fibrin clot lysis (T_50%_) by APAC (95±6.1 min) or SLK (145±7.1 min) was much longer than that by combined APAC+SLK (65±7.6 min) at the final concentration of 0.025 µM (APAC+SLK vs APAC, p<0.05; APAC+SLK vs SLK, p<0.01). Thus these low concentrations of a combination of both agents are likely to be more effective and less toxic when used therapeutically in vivo.

## Introduction

Stroke is the second leading cause of death worldwide [1], [2]. Approximately 80% of strokes are caused by focal cerebral ischemia due to arterial occlusion, whereas up to 20% are caused by intracerebral hemorrhages [3], [4]. In ischemic stroke, treatment options are limited. Therapeutic thrombolysis is restricted to the first few hours after onset [5]–[7], and the utility of current platelet aggregation inhibitors [8], [9], including α_IIb_β_3_ antagonists, is counterbalanced by the risk of intracerebral bleeding complications. Thus, there is a pressing need to develop better and safer therapeutic approaches with an improved benefit-to-risk ratio.

We have previously described a unique antiplatelet autoantibody in patients with HIV- or hepatitis C-related thrombocytopenia that recognizes platelet integrin GPIIIa49-66 epitope and induces complement-independent platelet fragmentation and death by generation of reactive oxygen species through the activation of 12-lipoxygenase and NADPH oxidase [10]–[14]. Subsequently, we identified a human single-chain fragment variable region (scFv) antibody (named A11), which induces comparable oxidative platelet fragmentation as the patient antibody [15]. To enhance its targeting, we produced a bifunctional A11-plasminogen first kringle-l agent (named SLK), which homes to newly deposited fibrin strands within and surrounding the platelet thrombus, reducing effects on non-activated circulating platelets [16]. This approach was successful for the clearance of preexisting arterial thrombus in murine models of ischemic stroke. Furthermore, we showed SLK to be associated with an even more modest drop in platelet count compared to A11 (11% versus 18%) [16]. In the current study we tested the hypothesis that synergistic administration of SLK with another GPIIIa49-66 targeting agent (A11) that homes to activated platelets will provide a better and safer therapeutic strategy for cerebral ischemia. We have developed an additional bifunctional platelet integrin GPIIIa49-66 agent (named APAC), and investigated its synergy with SLK for the dissolution of ex vivo platelet thrombus at low concentrations.

## Materials and Methods

### Materials

All reagents were obtained from Sigma (St. Louis, MO), unless otherwise designated. E.*coli* strains Rosseta, plasmid pET-29a and Ni-NTA agrose resin were from Novagen (Nottingham, UK). Restriction enzymes were obtained from New England Biolabs (Beverly, MA). Tomlinson human scFv monoclonal phage J library was kindly provided by MRC Geneservice (Cambridge, UK).

### Cloning, expression and purification of bifunctional scFv-A11-PAC-1 (APAC) reagent

The Tomlinson J phage Library was used to screen against a biotin conjugated GPIIIa49-66 peptide. Specific clones enriched for anti-GPIIIa49-66 Ab's were screened and one clone named A11 was selected for highest binding avidity, as described [15]. PAC-1 is an IgM-κ murine monoclonal antibody that, like fibrinogen, binds to α_IIb_β_3_ only on activated platelets. The binding of PAC-1 to activated platelets mainly depend on the RYD sequence within the H-CDR3 (heavy chain variable region) which mimics the RGD sequence in fibrinogen [17]. We commercially produced the cDNA from the heavy and light chain variable region. We have substituted the published RYD binding region with RGD for possible greater integrin binding. We next linked A11 to the heavy-light chain variable binding region of PAC-1 (APAC) with a (GSTSG)_3_SGSGI linker. The forward primer of PAC-1 (PACF) 5′- ACAAGTGGTGGATCTACTAGTGGCTCTGGATCCGGAATTCAGGTGCAGCTGAAGCAGTCA-3′ have three portions: the first 20 bp is the reverse complement sequence of part of the SCFC primer. The residual sequence encodes for the C-terminal half of the linker and the beginning of the PAC-1 domain. The backward primer (PACR) is 5′-TAGGATCCGCGGCCGCTTTTATTTCCAGCTT GGTCCC-3′. The 1548 bp ScFv-A11-Linker-PAC (APAC) was inserted into pET-29a to generate pET29a- APAC. *E.coli* Rosetta cells transformed with the expression vector pET29a-APAC were cultured in 1 L 2YT medium containing carbenicillin (50 µg/ml) and chloramphenicol (34 µg/ml) with shaking at 37°C until the OD (600 nm) was 0.4–0.6. Production was induced by the addition of 1 mM IPTG and the cells incubated at 37°C for 4 hours with shaking. Cells were then harvested and the pellets frozen at −20°C for storage. Refolding and purification steps were performed at 4°C as previously described [16].

### Platelet activation

Platelets from platelet-rich plasma (PRP) were activated by addition of 20 µM ADP (Helena Lab, Beaumont, TX), and then incubated for 20 minutes at room temperature with 1 µM APAC. Secondary staining was performed with monoclonal, FITC-labeled anti-His (6)-tag antibody (Dianova, Hamburg, Germany). The binding to the platelets was determined by flow cytometry using a FACScan (BD Biosciences, Mountain View, CA).

### Platelet particle assay

Gel-filtered human platelets or thrombin PAR-1 agonist TFLLRN-activated human platelets (100 µM TFLLRN) were stained with anti-CD61-FITC respectively, and then treated with various concentrations of APAC for 4 hours at 37°C. Fluorescent-labeled platelets/particles were measured by flow cytometry using a FACScan (BD Biosciences, Mountain View, CA) as previously described [13].

### Disaggregation and destruction of ex vivo human platelet aggregates

For collagen-induced aggregates, 1 µg/mL collagen (Helena Lab, Lubbock, TX) was incubated with human platelets for 1 hour at 37°C with intermittent shaking, followed by gravity sedimentation at room temperature for 0.5 h. The top 50% volume was removed and the remainder of the platelet aggregate suspension was added directly into Tyrode buffer, pH 7.4 with testing reagents. A11, SLK, APAC or SLK+APAC were added for various time intervals, and the remaining platelet/aggregate was enumerated as previously described [16].

### Platelet-rich fibrin clot lysis assay

Platelet-rich fibrin clots were formed as described [18]. Briefly, clotting was initiated by adding human thrombin (0.6 NIH unit/mL), CaCl_2_ (to 20 mM) and human fibrinogen (1 mg/mL) in human platelet. Each clot in the microtiter plate was incubated with PBS containing plasmin (1 pmol/well) and varied concentrations of control scFv Ab (13CG2), SLK, APAC, SLK+APAC. After 30 min the surface of each clot was washed gently with PBST (PBS with 0.1% Tween-20) to remove unbound plasmin and agents. The platelet-rich clot lysis process was monitored by measuring turbidity changes in the absorbance at 405 nm at 25°C using a microtiter plate reader operated in the kinetic mode. All readings reached the low plateau were taken as completion of clot lysis. Duplicate wells were prepared for each concentration of test agents in each experiment, and the experiment was repeated three times. T_50%_ represented the time to achieve a 50% lysis of the platelet-rich clot. It was calculated from the OD/time data.

### Statistics

Data are expressed as mean ±standard deviation (SD). One way analysis of variance (ANOVA) followed by Tukey's test for multiple comparisons. In the case of single comparisons, student's *t*-test was applied. *P*-values≤0.05 were considered statistically significant.

## Results

### Production and characterization of a bifunctional APAC capable of homing to active platelets

**Figure 1:**
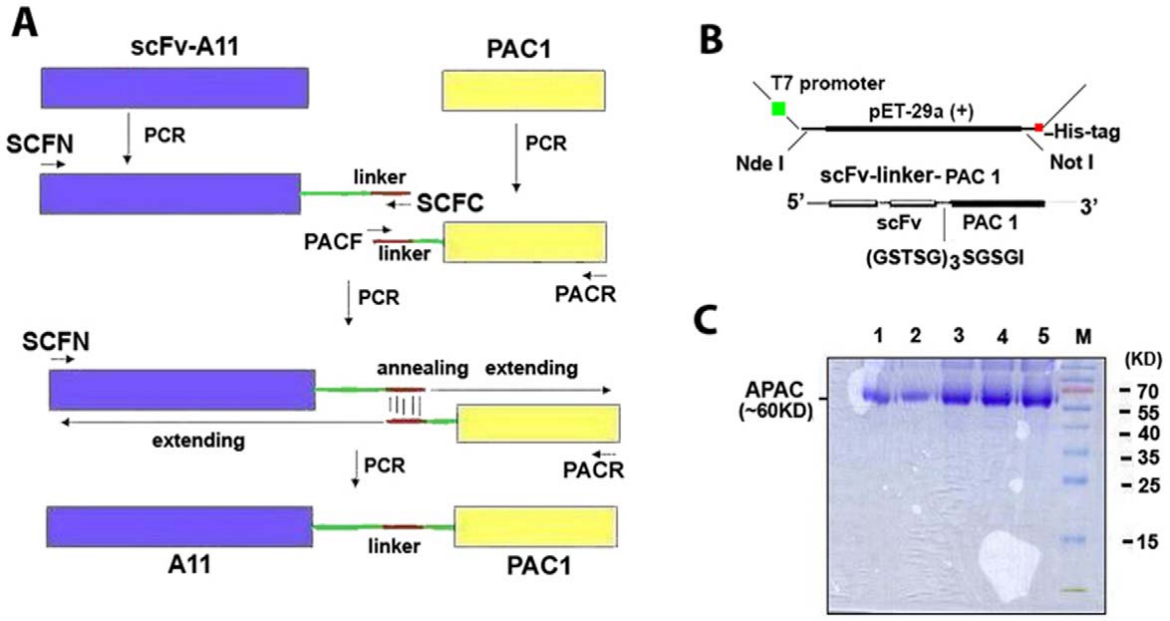
Generation of a bifunctional APAC capable of homing to active platelets. (A) Schematic diagram describing cloning strategy for the fusion construct. (B) Plasmid Construct. (C) 12% SDS-PAGE analysis of five aliquots of purified fusion protein (∼60 KD) with Ni-column following induction by 1 mM IPTG.

To enhance the protective effect of A11, we devised an approach to home A11 to activated platelets by coupled A11 with scFv-PAC-1 (named APAC) as previously reported for SLK [16]. Figure 1 demonstrates the rationale of the approach.

**Figure 2:**
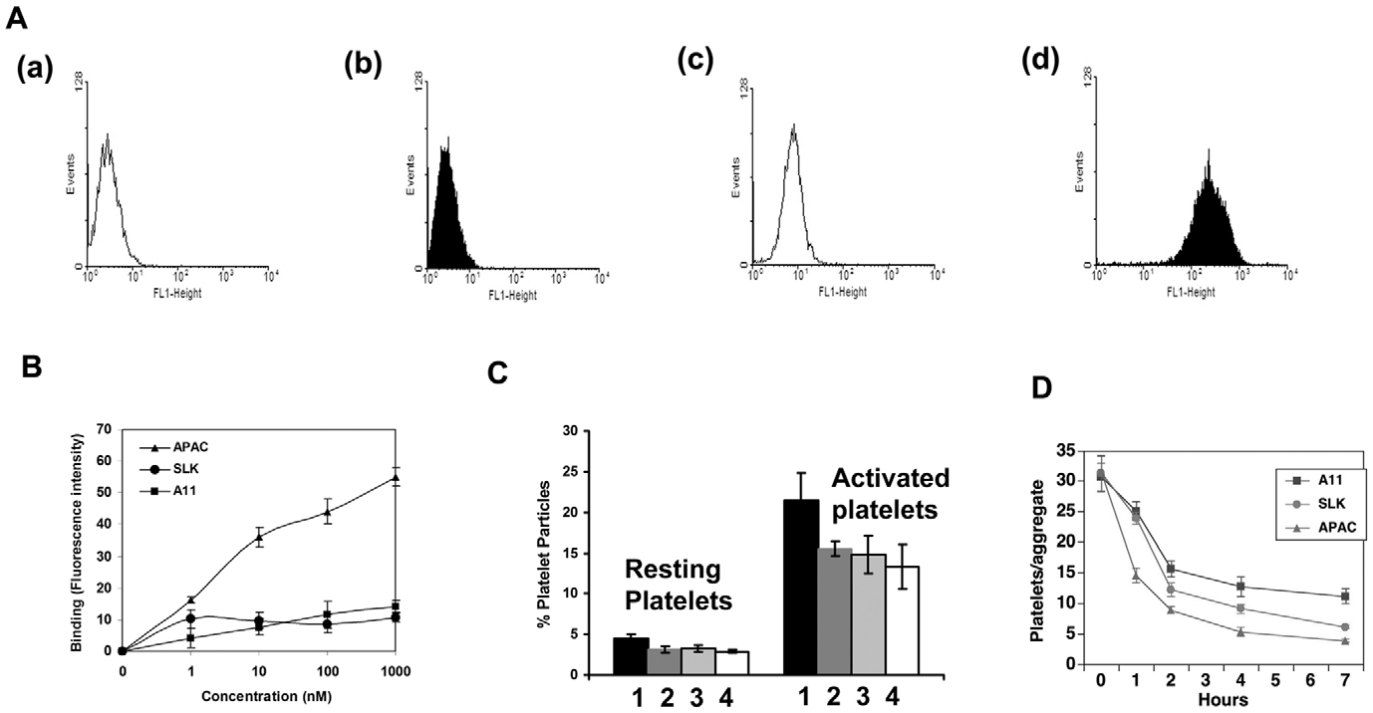
Characterization of a bifunctional APAC agent capable of homing to active platelets. (A) Binding assay of APAC to platelets. (a) resting mouse platelets; (b) ADP-stimulated mouse platelets; (c) resting human platelets; (d) ADP-stimulated human platelets, respectively. (B) Binding assay of APAC versus A11 to activated human platelets determined by flow cytometry. Data were presented as mean ±SD (n = 3). (C) Effect of APAC on platelet fragmentation. Bars labeled 2, 3 and 4 after 1 refer to serial doubling dilutions of 1∶2–1∶16 respectively (0.1 µM APAC), n = 4, SD is given. (D) Dissolution of ex vivo collagen-induced platelet aggregates with APAC. Data and SD are given for 3 separate experiments at 0.1 µM reagent in which each time point represents 5 measurements.

The specificity of APAC binding to activated platelets was determined by flow cytometry. Figure 2A demonstrates APAC preferably bound to ADP-activated human platelets, whereas it did not bind to human resting platelets or mouse platelets. Figure 2B demonstrates APAC binding to ADP-activated human platelets in a concentration-dependent manner, which is ∼4.8 fold more potent than parent A11 or SLK (which targets newly deposited fibrin strands surrounding the platelet thrombus).

We next investigated the effect of APAC on platelet fragmentation. As demonstrated by Figure 2C, APAC has a preferential effect on activated platelets pretreated with the thrombin PAR-1 agonist TFLLRN, particularly at low APAC concentrations (∼4.6-fold greater sensitivity). Notably, platelet fragmentation induced by APAC was inhibited by the peroxide inhibitors catalase and diphenylene iodonium (DPI), as previously reported for anti-GPIIIa49-66 Abs, indicating the requirement of an oxidation reaction for platelet fragmentation (data not shown).

Since APAC binds to human platelets, but not mouse platelets, we could not test the effect of APAC *in vivo* in a mouse model as we have previously reported. We therefore designed an ex vivo experiment with human platelets. Like SLK, a total of 0.1 µM APAC destroyed ex vivo platelet aggregates in an identical fashion with ∼85% destruction of platelet aggregates at 2 hours (Figure 2D).

### Synergy of APAC and SLK on ex vivo platelet aggregate dissolution and platelet-rich clot lysis

**Figure 3:**
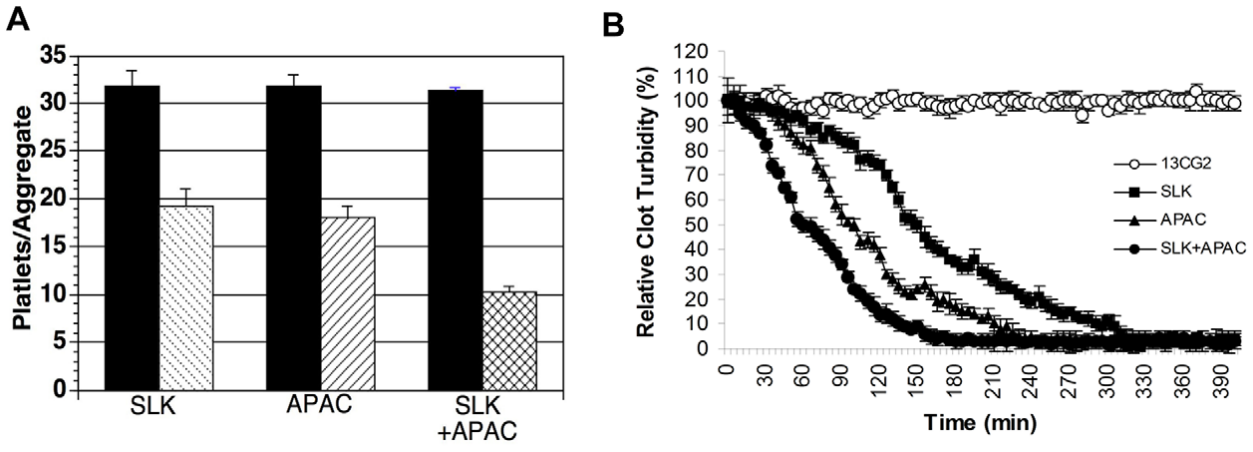
Synergy of APAC and SLK on ex vivo platelet aggregate dissolution and platelet-rich clot lysis. (A) Platelet aggregates were prepared as described above. Black bars refer to platelet aggregate size at zero time. The 3 companion hatched bars refer to platelet aggregate size at 2 hours. Concentration of SLK and APAC was at 0.025 µM. SLK+APAC double hatched bar refers to final SLK and APAC concentration at 0.025 µM each. (B) Platelet-rich clots were formed on the wells of ELISA plate. The clots were treated with 0.025 µM Ctl scFv (13CG2) or SLK or APAC or SLK+APAC. SLK+APAC refer to final SLK and APAC concentration at 0.025 µM each. The relative clot turbidity was calculated by detecting the decrease of the absorbance at OD405. The mean±SD. came from 3 separate experiments. Each time point represents 5 measurements.

Because bifunctional APAC binds to activated platelets and SLK binds fibrin surrounding the platelet thrombus, we designed an experiment to test synergy when both agents were utilized together at a low concentration. As hypothesized, platelet aggregate dissolution by the combination of SLK and APAC was ∼2 fold greater than either agent alone at 0.025 µM (Figure 3A). Similar results were obtained with a combination of SLK and APAC at the final concentrations of 0.05 or 0.1 µM (∼1.81-fold and 2.16-fold greater sensitivity, respectively; data not shown).

To further investigate synergy of SLK and APAC, we performed platelet-rich clot lysis experiments which partially simulate the physiologic condition. Figure 3B demonstrated the time required for 50% platelet-rich fibrin clot lysis (T_50%_) by APAC (95±6.1 min) or SLK(145±7.1 min) was much longer than that by APAC+SLK (65±7.6 min) at the final concentration of 0.025 µM (APAC+SLK vs APAC, p<0.05;APAC+SLK vs SLK, p<0.01).

## Discussion

Currently, the αIIbβ3 integrin has been utilized as a therapeutic target for thrombosis and occlusion due to coronary artery stenting [19]. A single-chain variable fragment antibody that specifically blocks the activated form of GPIIb/IIIa has been successfully produced [20]–[22]. In contrast to clinically used anti-platelet agents [23]–[26], none of which are conformation specific, scFvs specifically targeting activated platelets do not induce conformational changes in GPIIb/IIIa or outside-in signaling, thereby avoiding unwanted global activation of platelets [21]. The exclusive binding property of these scFvs to activated platelet-specific is mediated by RXD motifs in the heavy chain complementary-determining region (CDR) 3 of the antibodies [22]. While these blockers selectively recognize activated platelets, they allow the necessary physiological functions of platelets, including cell adhesion and spreading on immobilized fibrinogen [22]. Therefore, activation-specific GPIIb/IIIa blockade via human single-chain antibodies represents a promising novel strategy for antiplatelet therapy an enhanced benefit to risk ratio.

In the present study, we report a novel and effective therapeutic strategy for the clearance of pre-existing platelet thrombus by the synergistic administration of a low concentration of two bifunctional antibodies against β3 integrin. Compared with other anti- αIIbβ_3_ antibodies, APAC has a different mechanism of action and has distinct properties that may be of clinical value. It directly dissolves the already-formed platelet thrombus by inducing oxidative platelet fragmentation. The selectivity of APAC for activated platelets is high. The 4.6 fold difference between the fragmentations of activated platelets versus resting platelets prevents the possible fragmentation of non-activated circulating platelets. Since APAC binds to human platelets but not mouse platelets, we could not test the effect of APAC in vivo using mouse models. However, current data demonstrates APAC is more effective than SLK on ex vivo platelet aggregate dissolution (Figure 2D) and platelet-rich clot lysis (Figure 3B). We have previously shown that SLK was effective for clearance of pre-existing arterial thrombus in murine models of ischemic stroke [16]. It has no effect on platelet function and minimal effect on platelet count (∼11% decrease) [16]. We therefore infer that APAC would probably provide better protection with a low bleeding risk in vivo compared to SLK. Moreover, this study clearly demonstrates that combinations of both agents at low concentrations (0.025 µM) are more effective than either agent alone for the dissolution of existing platelet thrombus. Thus in potential future clinical applications, the synergistic administration of both agents will likely be more effective and less toxic in vivo at these low concentrations. We hope that in the future our preclinical data will lead to Phase I testing in humans, in whom we expect APAC to provide a superior treatment option for ischemic stroke.

These findings provide a proof-of-principle for the development of a novel approach to dissolve pre-existing arterial thrombosis by the synergistic administration of low concentrations of two bifunctional GPIIIa49-66 ligands targeting different part of the platelet thrombus, which will contribute to preventing ischemic events as well as likely having a high benefit-to-risk ratio.
